# Relationship between relative fat mass and low-carbohydrate diet scores and sleep disorders in United States: a real-world cross-sectional study

**DOI:** 10.3389/fnut.2024.1500934

**Published:** 2024-10-24

**Authors:** Cheng Cao, Keyi Yu, Fuquan Lin, Aie Xu, Miaoni Zhou

**Affiliations:** ^1^Department of Dermatology, Hangzhou Third Hospital Affiliated to Zhejiang Chinese Medical University, Hangzhou, China; ^2^School of Basic Medical Sciences, Zhejiang Chinese Medical University, Hangzhou, China

**Keywords:** sleep disorder, RFM index, obesity, National Health and Nutrition Examination Survey, low-carbohydrate-diet score

## Abstract

**Objective:**

To investigate the relationship between relative fat mass (RFM) and low-carbohydrate diet (LCD) scores and sleep disorders in the U.S. population.

**Methods:**

Data were collected from the National Health and Nutrition Examination Survey (NHANES) conducted between 2005 and 2014. A total of 5,394 respondents participated in the study. Univariate and multivariate linear regression analyses were used to investigate the relationship between RFM and LCD scores, and univariate and multivariate logistic regression analyses were used to investigate the relationship between RFM and LCD scores and sleep disorders. Restricted cubic spline (RCS) analyses were conducted to test for nonlinear associations between RFM and LCD scores and sleep disorders.

**Results:**

A total of 5,394 participants were included in the statistical analysis, including 5,080 healthy participants and 314 with sleep disorders. Univariate and multivariate linear regression showed a bivariate positive correlation between RFM and LCD scores (*p* < 0.05), and logistic regression analysis showed a significant positive correlation between RFM (95% CI: 1.02–1.07, *p* = 0.005) LCD scores (95% CI: 1.00–1.03, *p* = 0.044) and sleep disturbances. Subgroup analyses showed robust effects of RFM and LCD score on sleep disorders.

**Conclusion:**

RFM was positively and bi-directionally associated with LCD scores, both of which resulted as risk factors for sleep disorders. This study emphasizes that an LCD and lowering RFM can prevent and ameliorate the risk of sleep disorders.

## 1 Introduction

Sleep is essential for physical and mental health. However, the high prevalence of sleep disorders is becoming a serious public health problem today, affecting 30–50% of the global population. In addition, sleep disorders and poor sleep quality have been associated with many chronic diseases, including diabetes, hypertension, depression, and obesity (1). Over the past few decades, a large body of scientific work has investigated the potential association between sleep duration and obesity in childhood. The findings suggest that sleep deprivation is associated with a significant increase in caloric intake (2). There is substantial evidence that dietary factors and obesity significantly influence the prevalence of sleep disorders and that LCDs and effective weight loss can significantly benefit people (3).

Obesity is usually defined as excessive fat storage, and obesity has significant health implications (4). BMI was first developed by a Belgian mathematician 200 years ago and is currently the most widely used tool for diagnosing obesity (5). Body mass index (BMI) is a common measure of obesity; however, BMI is not sufficient to assess obesity because people with the same BMI may have different body types and fat distribution (6). In addition, BMI does not distinguish abdominal fat distribution well. Researchers have developed a new indicator of obesity to more precisely assess fat mass, RFM, which includes waist circumference (WC) and height (7). RFM is more accurate than BMI in determining a person’s body fat percentage. RFM has been reported to be associated with several diseases, including venous thromboembolism, coronary heart disease, hypertension, and type 2 diabetes mellitus (8, 9). The recent study reported a stronger association between RFM and obesity compared to BMI and WC, which remained robust even when considering the influence of depression (10).

When it comes to the impact of obesity, available research shows that the prevalence of obesity has increased significantly worldwide over the past decade. A smaller but equally important increase has been observed in obesity-related comorbidities (type 2 diabetes, cardiovascular disease, cancer), which are becoming a real burden on healthcare systems. In addition to the most common complications of obesity, sleep disorders have recently become one of the obesity-related complications (11). In their study, Hargens et al. (12) pointed out that another critical mechanism linking obesity to sleep disorders is diet quality, which possibly acts through the effects of nutrients on inflammatory and/or hormonal responses that involve hunger-satiety mechanisms, energy metabolism, and circadian rhythms. A high-fat/high-carbohydrate diet has been reported to cause obesity and if untreated leads to steatohepatitis (13). In turn, increasing evidence has shown that sleep disorders may increase the risk of obesity and metabolic diseases (14).

Recently, the LCD score, characterized by lower carbohydrate intake and higher protein and fat intake, has been proposed (15). The LCD score is gaining widespread attention as an effective modality for weight loss and obesity prevention (15, 16). Previous studies have shown that LCD scores are associated with many aspects of health, such as diabetes, progression of coronary artery calcification, and psychological disorders (15, 17). In their study, Sangsefidi et al. reported that LCD scores may be associated with lower odds of metabolic syndrome in Iranian adults, according to a cross-sectional study (18). Lower low-carb diets have been demonstrated in recent research to improve weight loss, cardiovascular health, and mortality risk in middle-aged and older persons (19).

However, to the best of our knowledge, only a few studies have explored the association of LCD scores with RFM index and sleep disorders in the U.S. population. Herein, we revisited the relationship between RFM and LCD scores and sleep disorders, which may contribute to developing future approaches for preventing or treating sleep disorders. We investigated the association of RFM and LCD scores with sleep disorders by analyzing data from 5,394 participants in NHANES from 2005 to 2014.

## 2 Methods

### 2.1 Study population and design

**Figure 1:**
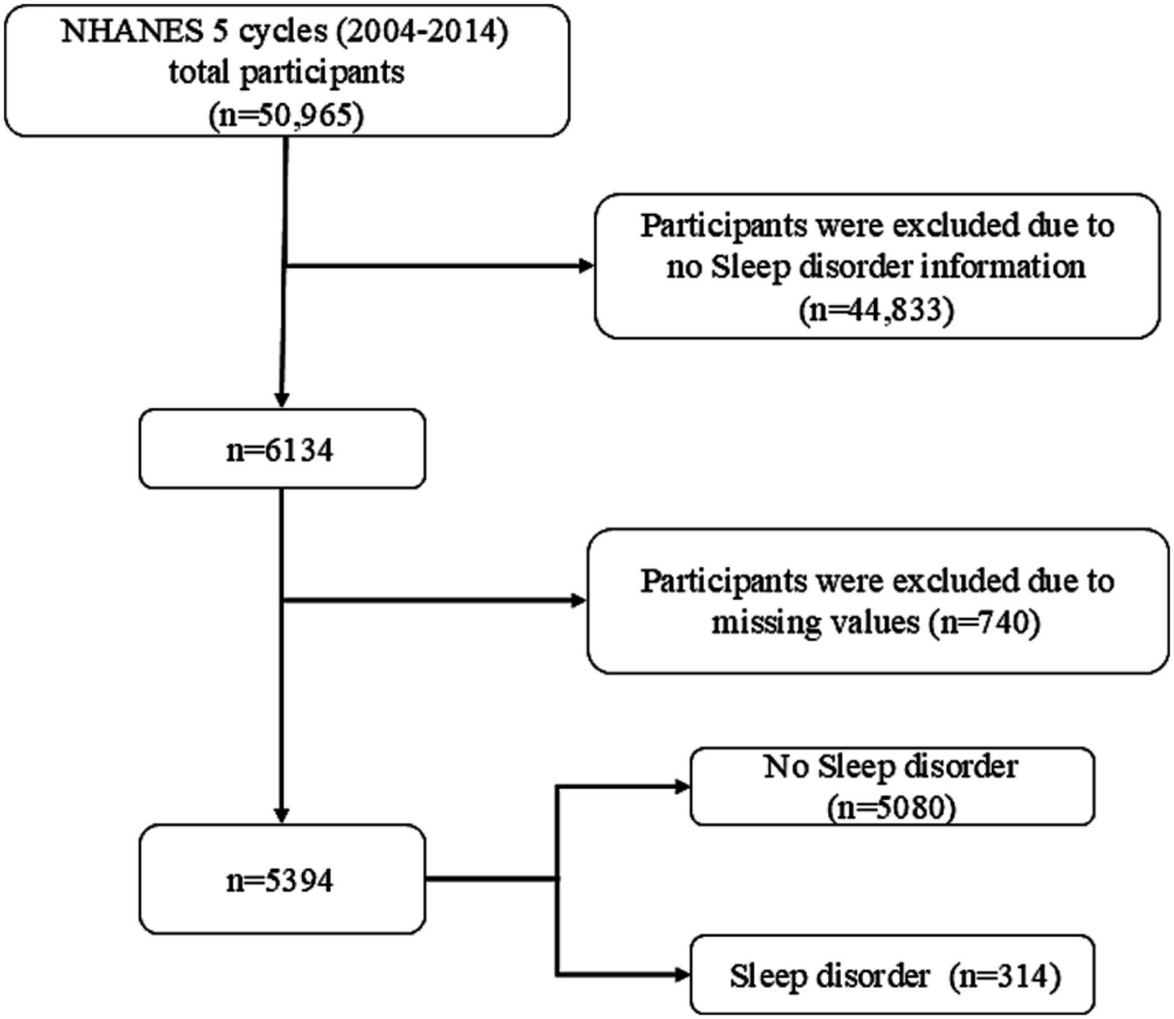
Flow chart of participants selection. NHANES, National Health and Nutrition Examination Survey.

The Centers for Disease Control and Prevention (CDC) collected five data cycles (2005–2014) from NHANES, a multistage, stratified, large, nationally representative study of the U.S. population that provides detailed information on study design, interviews, and demographics. In a sample of 50,965 adults, those who did not provide information on sleep disorders, BMI, height, weight, waist circumference, dietary information, or other variables were excluded (Figure 1).

The protocol was approved by the Ethical Review Board of the National Center for Health Statistics, and informed consent was obtained from participants.

### 2.2 Calculation of the RFM and LCD score

Physical measurements of waist circumference and body weight were taken by trained health technicians in a mobile testing center (MEC) under controlled conditions. As an exposure variable, the RFM results of each participant were rounded to two decimal places. In our analysis, RFM was used as a continuous variable. It was calculated from waist circumference, height, and sex, using the following formula: RFM = 64 – (height 20 ×/waist circumference) + (12 × sex), sex: female = 1, male = 0 (7).

LCD scores were calculated from dietary data from the first 24-h dietary recall interview used to calculate fat, protein, carbohydrate, and energy intake. LCD scores were calculated based on a combined assessment of fat, protein, and carbohydrate (16). Intake per gram of fat, protein, and carbohydrate was first converted to kilocalories (conversion ratios of 1:9, 1:4, and 1:4, respectively), after which the percentage of carbohydrate (kilocalories) and total energy, the percentage of protein (kilocalories) and total energy, and the percentage of fat (kilocalories) and total energy were calculated. For fats and proteins, the percent intake was scored as a maximum of 10 points and a minimum of 0 points. For carbohydrates, the lowest percentage intake scored 10 points, and the highest scored 0 points.

### 2.3 Diagnosis of sleep disorder

Participants were identified by the questionnaire item “Ever told by a doctor to have a sleep disorder?” under the “Sleep Disorders” section. Only those patients who answered that they had been told they had or did not have a history of sleep disorder were included in the study.

### 2.4 Assessment of other variables

NHANES collects sociodemographic information through structured data. Demographic covariates considered in this study included age, gender, race, smoking status, drinking history, education, and annual household income. Waist circumference, weight, and height were measured by trained health technicians in accordance with the anthropometric procedures manual. BMI was calculated as weight (kg) divided by the square of height (m^2^). We defined smoking history as “<100 cigarettes in a lifetime” and alcohol consumption history as “at least 12 alcoholic drinks per year.”

### 2.5 Statistical analyses

Data were statistically analyzed using R open-source software version 4.2.2. The characteristics of each participant were analyzed descriptively. Means and standard deviations (SD) were reported for continuous variables, as well as percentages for categorical variables. Univariate and multivariate linear regression analyses were used to determine the relationship between RFM and LCD scores, and the relationship between RFM and LCD scores and sleep disorders was explored using univariate and multivariate logistic regression models. RCS analysis based on multivariate logistic analysis in Model 3 and Model 6 was used to assess the nonlinear association between RFM and LCD scores and sleep disorders. The weights of all survey samples were considered in data analysis. *P*-value <0.05 was considered statistically significant.

## 3 Results

### 3.1 Demographic characteristics of participants

A total of 50,965 individuals participated in NHANES between 2005 and 2014 (Figure 1). Participants who did not provide necessary information such as waist circumference, height, weight, and dietary information were excluded, yielding a sample of 5,394 participants. The study included 5,080 healthy participants and 314 participants with sleep disorders. Table 1 shows the general characteristics of the study population, grouped by the presence or absence of sleep disorders. It was found that patients with sleep disorders were more likely to be married, middle-aged or elderly, obese, and have a history of smoking compared to the general population. The mean (SE) RFM of the study population was 34.23 (9.08) and the mean (SE) LCD score was 9.89 (7.48), and patients with sleep disorders possessed higher RFM (*p* = 0.001) and LCD (*p* = 0.025) scores than the normal population without sleep disorders (Table 1).

**Table 1:** Basic characteristics of participants by whether they had sleep disorders or not.

Variables	Total (*n* = 5,394)	No- sleep disorders (*n* = 5,080)	With- sleep disorders (*n* = 314)	*p*
Age, Mean ± SD	41.70 (20.50)	44.03 (18.04)	49.43 (15.56)	<0.001^***^
Weight, Mean ± SD	79.40 (20.78)	80.03 (20.42)	92.58 (23.32)	<0.001^***^
Waist, Mean ± SD	95.89 (16.53)	95.87 (15.96)	107.13 (18.15)	<0.001^***^
Height, Mean ± SD	167.92 (10.10)	169.00 (10.01)	169.74 (10.16)	0.315
BMI, Mean ± SD	28.08 (6.62)	27.94 (6.45)	32.12 (7.65)	<0.001^***^
RFM, Mean ± SD	34.23 (9.08)	34.11 (8.50)	37.25 (8.06)	0.001^**^
Energy, Mean ± SD	2211.66 (1056.45)	2249.29 (1054.26)	2250.88 (1131.84)	0.984
Protein, Mean ± SD	83.60 (45.01)	85.80 (44.95)	87.45 (45.57)	0.426
Carb, Mean ± SD	272.10 (137.24)	267.14 (134.64)	259.68 (142.82)	0.287
Fat, Mean ± SD	83.30 (47.77)	84.74 (47.82)	86.95 (50.73)	0.589
LCD Score, Mean ± SD	9.89 (7.48)	10.55 (7.57)	11.60 (7.62)	0.025^*^
Race, *n* (%)				0.138
Mexican American	1,211 (22.45)	1,160 (8.5)	51 (5.6)	
Non-Hispanic Black	1,329 (24.64)	1,265 (11.9)	64 (8.6)	
Non-Hispanic White	2,460 (45.61)	2,284 (71.6)	176 (77.4)	
Other Hispanic	171 (3.17)	163 (2.9)	8 (2.2)	
Other Race	223 (4.13)	208 (5.2)	15 (6.2)	
Gender, *n* (%)				0.402
Female	2,796 (51.84)	2,647 (52.1)	149 (48.5)	
Male	2,598 (48.16)	2,433 (47.9)	165 (51.5)	
Marital status, *n* (%)				<0.001
Divorced	412 (7.64)	374 (10.2)	38 (14.5)	
Living with partner	421 (7.80)	400 (8.2)	21 (6.0)	
Married	2,435 (45.14)	2,259 (51.9)	176 (61.0)	
Never married	1,641 (30.42)	1,590 (21.9)	51 (12.4)	
Separated	145 (2.69)	132 (2.1)	13 (3.9)	
Widowed	340 (6.30)	325 (5.7)	15 (3.1)	
Education, *n* (%)				0.034
Below high school	1883 (34.91)	1799 (20.4)	84 (17.3)	
High school	1,243 (23.04)	1,165 (25.2)	78 (21.2)	
Over high school	2,268 (42.05)	2,116 (54.5)	152 (61.5)	
Income, *n* (%)				0.053
< 20,000	1,310 (24.29)	1,221 (16.3)	89 (22.2)	
> =20,000	4,084 (75.71)	3,859 (83.7)	225 (77.8)	
Smoke, *n* (%)				0.022^**^
No	3,338 (61.88)	3,187 (55.0)	151 (46.8)	
Yes	2056 (38.12)	1893 (45.0)	163 (53.2)	
Alcohol, *n* (%)				0.987
No	2,520 (46.72)	2,391 (33.9)	129 (33.9)	
Yes	2,874 (53.28)	2,689 (68.1)	185 (68.1)	

*t*, *t*-test; *χ*^2^, Chi-square test; SD, standard deviation. Values are presented as mean ± SD for continuous variables, and *p*-value was calculated by the weighted linear regression. Values are presented as percent (%) for categorical variables, and *p*-value was calculated by weighted chi-square test.

BMI, body mass index; RFM, relative fat mass; LCD, low-carbohydrate-diet.*p < 0.05, **p < 0.01, ***p  < 0.001.

### 3.2 Association between RFM, LCD score and sleep disorder

#### 3.2.1 Association between RFM and LCD score

Univariate linear regression was used to analyze the correlation between RFM and LCD score, revealing that RFM (95% CI: 1.01–1.06, *p* = 0.012) was positively correlated with LCD score. After the covariates of age, level of education, annual household income, smoking history, and history of alcohol consumption were combined in multivariate linear regression, RFM (95% CI: 1.02–1.07, *p* = 0.005, Table 2) remained significantly positively associated with LCD score, and the inclusion of multiple covariates strengthened this association.

**Table 2:** Correlation analysis between RFM and LCD score.

LCD score			
Model	Characteristic	OR (95% CI)	*p*
Model 1	RFM	1.03 [1.01, 1.06]	0.012^*^
Model 2	RFM	1.04 [1.02, 1.07]	0.005^**^
RFM			
Model 3	LCD score	1.04 [1.01, 1.07]	0.016^*^
Model 4	LCD score	1.05 [1.02, 1.09]	0.006^**^

Model 1, No covariates were adjusted. Model 2, Age, Marital status, educational level, income, smoking status and drinking alcohol status were adjusted; Model 3, No covariates were adjusted. Model 4, Age, Marital status, educational level, income, smoking status and drinking alcohol status were adjusted.

OR, Odds Ratio; CI, Confidence Interval; RFM, relative fat mass; LCD, low-carbohydrate-diet.*p < 0.05, **p < 0.01, ***p  < 0.001.

In turn, we also found that LCD score (95% CI: 1.01–1.07, *p* = 0.016) was positively associated with RFM, and after multivariate linear regression incorporating the covariates of age, education level, annual household income, smoking history, and drinking history, LCD score (95% CI: 1.02–1.09, *p* = 0.006, Table 2) remained significantly positively correlated with RFM, and this correlation was also strengthened with the inclusion of multiple covariates. This result clearly confirmed that obese people prefer high carbohydrate diets, which also promote obesity.

#### 3.2.2 Univariate logistics regression analysis of the association between RFM, LCD score, and sleep disorder

Based on baseline demographic information, univariate logistic analysis was used to observe the relationship between indicators with significant differences (age, weight, waist circumference, BMI, RFM, LCD score, smoke) and episodes of sleep disorders in the two participant groups. Our investigation found that age (95% CI: 1.01–1.02, *p* < 0.001), weight (95% CI: 1.02–1.03, *p* < 0.001), waist circumference (95% CI: 1.03–1.05, *p* < 0.001), BMI (95% CI: 1.07–1.11, *p* < 0.001), RFM (95% CI: 1.03–1.07, *p* < 0.001), never been married (95% CI: 0.20–0.78, *p* = 0.013), and LCD score (95% CI: 1.01–1.03, *p* = 0.003) were positively correlated with sleep disturbance. Patients with a history of smoking (95% CI: 1.11–1.78, *p* = 0.002, Table 3) were at a higher risk of developing sleep disorders than the healthy population.

**Table 3:** Correlation analysis between clinical features and sleep disorder in participants.

Variables	*p*	OR (95% CI)
Age	<0.001^***^	1.02 [1.01, 1.02]
Weight	<0.001^***^	1.03 [1.02, 1.03]
Waist	<0.001^***^	1.04 [1.03, 1.05]
BMI	<0.001^***^	1.09 [1.07, 1.11]
RFM	<0.001^***^	1.05 [1.03, 1.07]
LCD score	0.003^**^	1.02 [1.01, 1.03]
Marital status		
Divorced		1.00 (Reference)
Living with partner	0.175	0.52 [0.19, 1.42]
Married	0.443	0.81 [0.45, 1.45]
Never married	0.013^*^	0.40 [0.20, 0.78]
Separated	0.360	1.33 [0.68, 2.59]
Widowed	0.051	0.38 [0.15, 1.00]
Education		
Below high school		1.00 (Reference)
High school	0.977	0.99 [0.68, 1.45]
Over high school	0.052	1.33 [1.00, 1.78]
Smoke		
No		1.00 (Reference)
Yes	0.002^**^	1.45 [1.17, 1.78]

Univariate logistics regression analysis with psoriasis as the dependent variable.

OR, Odds Ratio; CI, Confidence Interval; BMI, body mass index; RFM, relative fat mass; LCD, low-carbohydrate-diet.*p < 0.05, **p < 0.01, ***p  < 0.001.

#### 3.2.3 Multivariable logistics regression analysis of the association between RFM, LCD score, and sleep disorder

As shown in the table, we constructed six logistic regression models to analyze the relationship between RFM, LCD score, and sleep disorders in the U.S. population. Models 1 and 4 were crude models unadjusted for covariates. Models 2 and 4 were adjusted for age, gender, and race. Model 3 was adjusted for age, sex, race, LCD score, education, annual household income, smoking history, and drinking history, and Model 6 was adjusted for age, sex, race, RFM, education, annual household income, smoking history, and drinking history. We found that RFM and LCD scores were positively associated with the risk of sleep disorders in all models, and covariate-adjusted Model 3 (95% CI: 1.02–1.07, *p* = 0.005) was significantly and positively associated with sleep disorders with Model 6 (95% CI: 1.00–1.03, *p* = 0.044, Table 4). Our investigation suggests that high levels of RFM with LCD score may be an independent risk factor for sleep disorders. After matching, the significant association between high levels of RFM with LCD score and increased risk of sleep disorders remained in all three models.

**Table 4:** Association of RFM and LCD score with psoriasis.

Model	Characteristic	OR (95% CI)	*p*
Model 1	RFM	1.04 [1.02, 1.07]	<0.001^***^
Model 2	RFM	1.04 [1.02, 1.06]	0.003^**^
Model 3	RFM	1.04 [1.02, 1.07]	0.005^**^
Model 4	LCD	1.02 [1.01, 1.03]	0.003^**^
Model 5	LCD	1.02 [1.00, 1.03]	0.030^*^
Model 6	LCD	1.02 [1.00, 1.03]	0.044^*^

Model 1, No covariates were adjusted. Model 2, Age was adjusted. Model 3, Age, gender, race, Marital status, educational level, income, smoking status and drinking alcohol status were adjusted; Model 4, No covariates were adjusted. Model 5, Age was adjusted. Model 6, Age, gender, race, Marital status, educational level, income, smoking status and drinking alcohol status were adjusted.

OR, Odds Ratio; CI, Confidence Interval.*p < 0.05, **p < 0.01, ***p  < 0.001.

#### 3.2.4 Subgroup analysis

**Figure 2:**
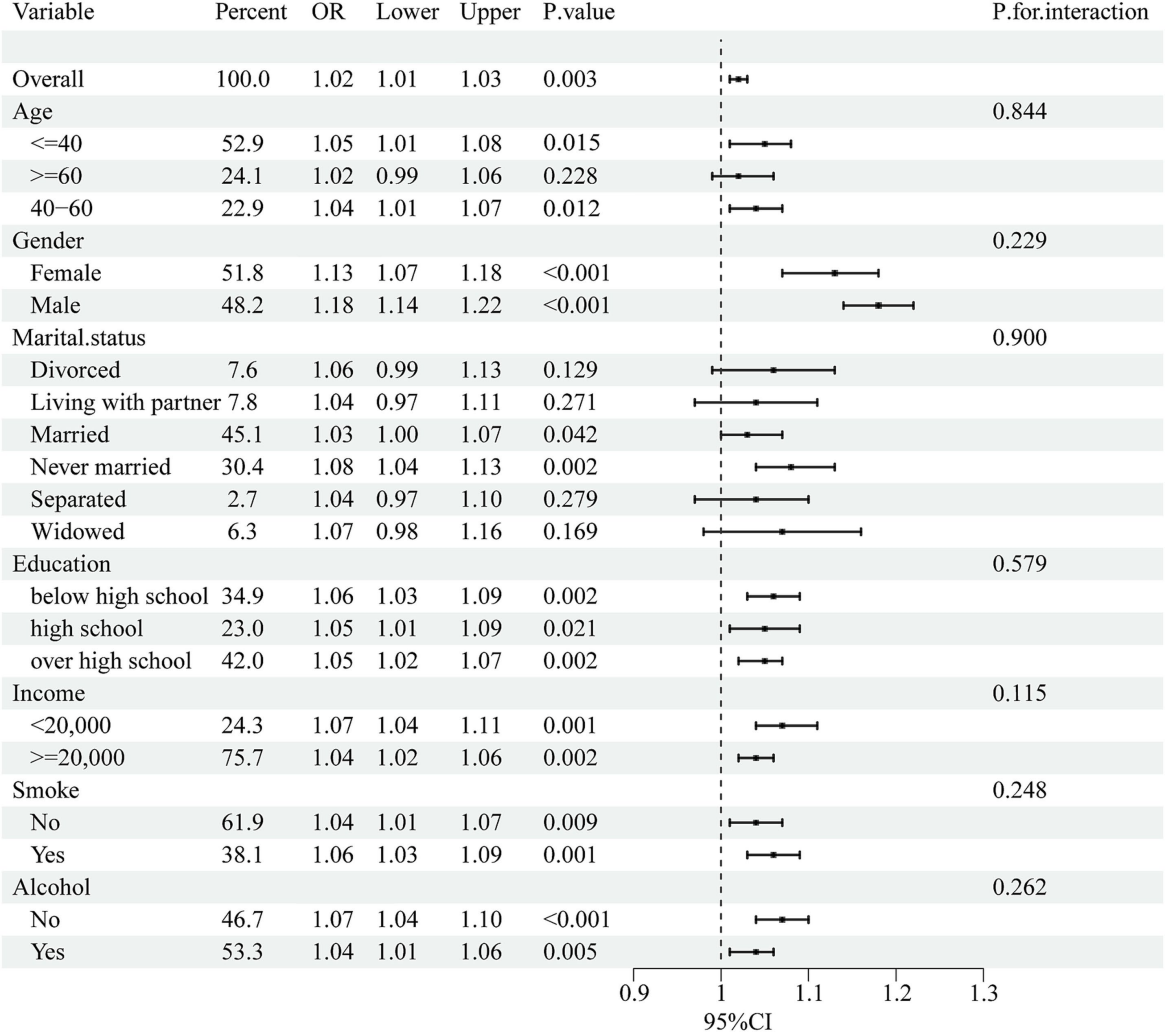
Subgroup analysis of the association between RFM and sleep disorders. Univariate logistic regression was used for subgroup analysis.

**Figure 3:**
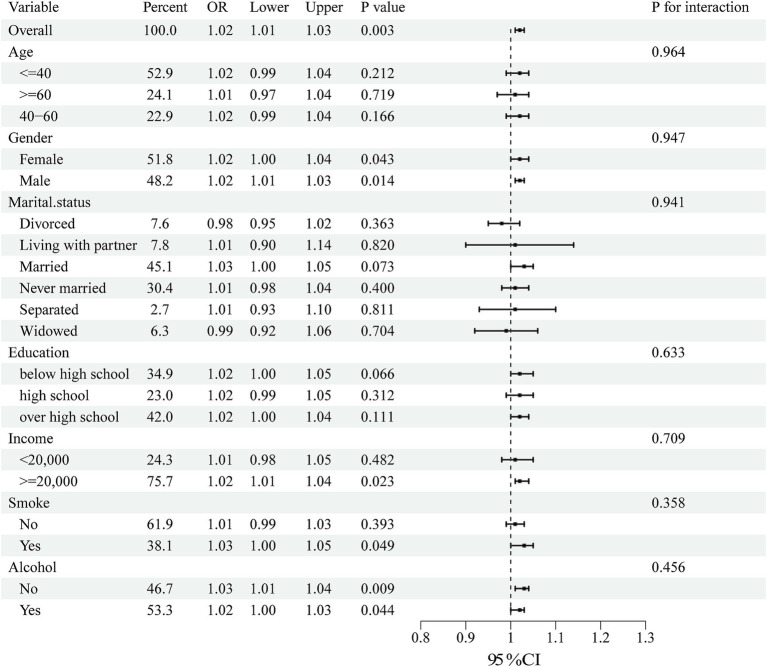
Subgroup analysis of the association between LCD score and sleep disorders. Univariate logistic regression was used for subgroup analysis.

Subgroup analyses were performed to assess the consistency of correlations between RFM and LCD scores and sleep disorders in different populations. The interactions between gender, age, marital status, education, annual household income, and smoking or drinking status were tested. Notably, subgroup analyses (Figures 2, 3) showed stable correlations between RFM and LCD scores and the occurrence of sleep disorders. Gender, age, education, educational level, annual household income, and smoking or drinking status had no significant effect on this correlation (*p* < 0.05).

#### 3.2.5 Nonlinear associations between RFM, LCD score, and the risk of sleep disorder

**Figure 4:**
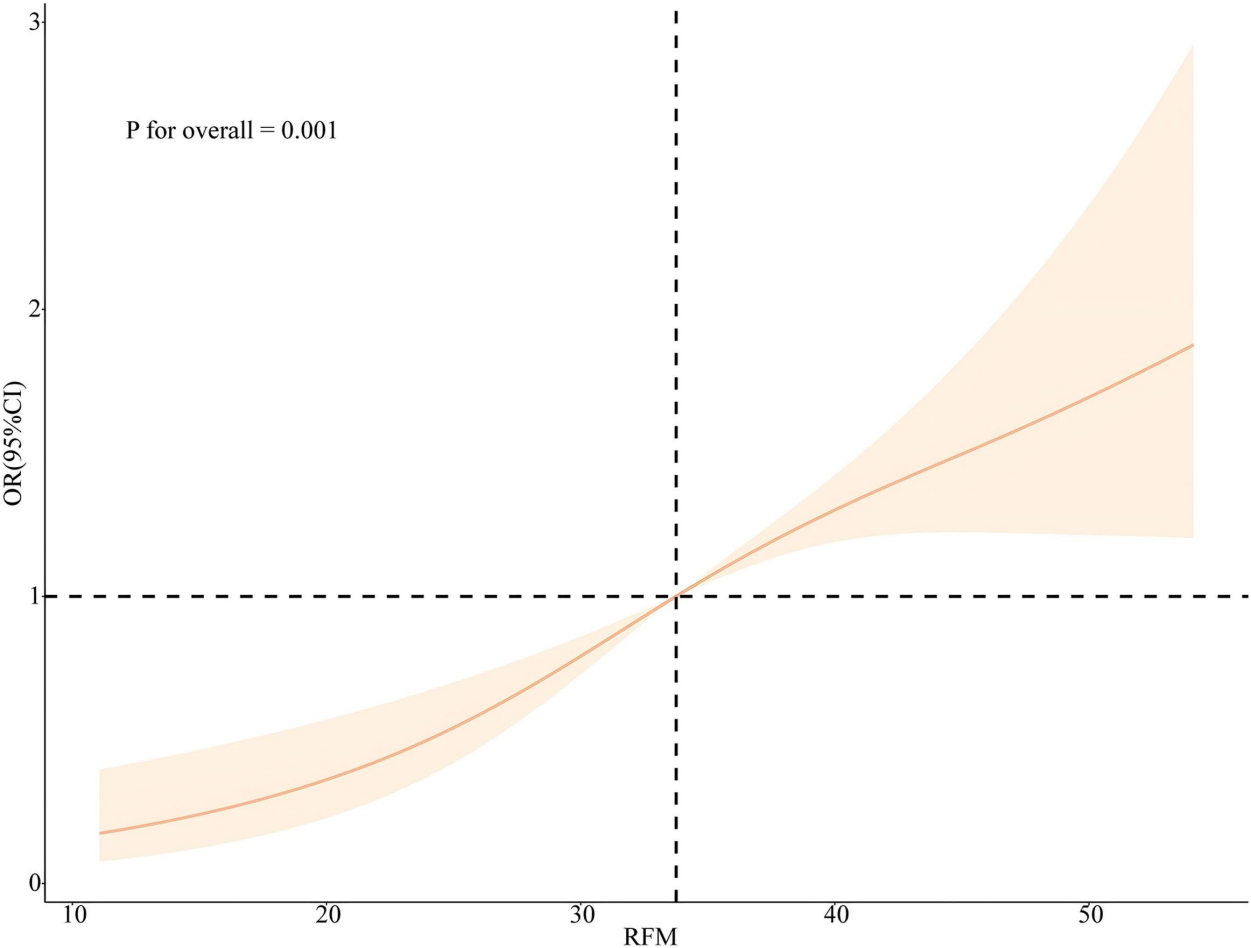
Nonlinear associations between RFM and the risk of sleep disorders legend: RCS based on Model 3.

**Figure 5:**
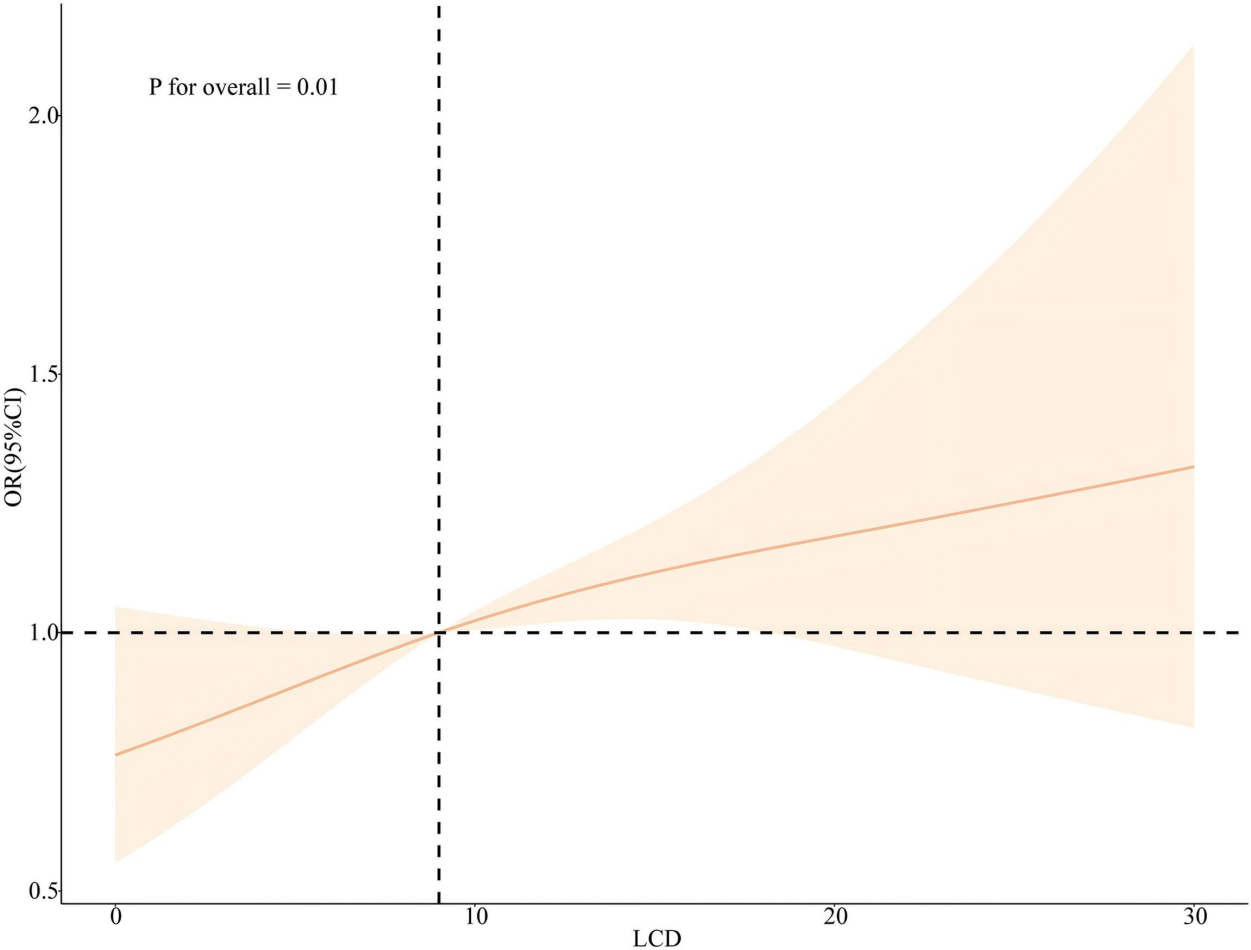
Nonlinear associations between LCD score and the risk of sleep disorders legend: RCS based on Model 6.

Plotting the RCS according to Model 3 and Model 6 visually depicts the relationship between RFM and LCD score with sleep disorder. As shown in Figures 4, 5, the RCS curves suggested an effect model with a significant relationship between RFM (*p* for overall = 0.001, *p* for nonlinear = 0.07) and LCD score (*p* for overall = 0.01, *p* for nonlinear = 0.51) and sleep disorder, although this effect seemed to be more skewed toward linearity than nonlinearity.

## 4 Discussion

The primary objective of this study was to assess the correlation between RFM and LCD scores and sleep disorders. We used NHANES data from 2005 to 2014 to investigate the relationship between RFM and LCD scores and sleep disorders in the U.S. national population. Our results showed that RFM and LCD score levels differed significantly between patients with sleep disorders and the normal population and that there was a positive correlation between RFM and LCD scores and sleep disorders. Univariate and multifactorial linear regression analyses showed that RFM and LCD scores were significantly and positively correlated with each other, which could explain why obese populations were more inclined to high-carbohydrate diets that can also cause obesity. Subsequent univariate and multivariate logistic regression analyses revealed that RFM and LCD scores may be independent risk factors for sleep disorders, and this correlation remained significant after adjusting for age, gender, race, marital status, education, annual household income, and smoking and drinking status. Subgroup analyses further showed that the correlation between RFM and LCD score and sleep disorders was stable regardless of age, gender, marital status, education, annual household income, and smoking and drinking status. In addition, there was no nonlinear association between RFM and LCD scores and sleep disorders analyzed by RCS.

The pathogenesis of sleep disorders is complex, and understanding the risk factors is necessary for effective prevention and treatment. Previous reports have identified obesity as one of the most important risk factors for sleep disorders, where a 6-unit increase in BMI resulted in a 4-fold increased risk of obstructive sleep apnea, which is characterized by recurrent narrowing and closure of the upper airway and can lead to intermittent hemoglobin oxygen desaturation, sleep fragmentation and daytime sleepiness (20–22). Obstructive sleep apnea syndrome (OSAS) is a highly prevalent sleep-related breathing disorder. It is characterized by hypopnea and apnea in ventilation, which cause intermittent hypoxia (IH) and lead to a number of other physiological effects. These include blood hypoxemia, hypercapnia, fragmented sleep, recurrent nocturnal arousals, enhanced respiratory effort, and increased sympathetic nerve activity (23). The risk of obstructive sleep apnoea syndrome (OSAS) is positively correlated with body mass index (BMI). As BMI increases, so too does the risk of OSAS, which is likely related to upper airway narrowing caused by excess fat tissue. Obesity can also induce a decrease in vital capacity, an imbalance in the ventilation-perfusion ratio, and limitations of lung and chest wall movement. Consequently, the countries with the highest prevalence of OSAS are those with high rates of obesity, and thus, the incidence of OSAS increases with increasing levels of obesity (24). However, while BMI is a commonly used indicator of obesity, it cannot accurately assess visceral fat, has limited ability to differentiate between lean body mass and adiposity, and its correlation with adiposity varies by biological sex, age, race, and disease (25). RFM is an emerging metric used for estimating total body fat percentage based on waist circumference, height, and biological sex. It is also a more accurate estimate of total body fat percentage than BMI (7). Another critical mechanism linking obesity to sleep disorders is dietary (12). Indeed, dietary fatty acids have been reported to be involved in sleep regulation through their dynamic effects on biochemical compounds, including complex lipids, prostaglandins, neurotransmitters, and amino acids, which are essential in initiating and maintaining sleep. Indeed, poor dietary quality (characterized by high-fat and low-fiber intake) has been associated with sleep disorders (26, 27). Notably, in the study conducted by Dragana Rogulja et al., it was observed that peptone supplementation led to an increase in CCHa1 levels within Drosophila gut cells, subsequently facilitating the suppression of sensory arousal during sleep and promoting longer periods of deep sleep among the flies (28). Overindulging in a high-fructose hypercaloric diet has been linked to elevated cortisol excretion in the urine and a greater sleeping metabolic rate when compared to an equivalent overfeeding diet where the carbs originate from whole-wheat items (29). The function of the dietary carbohydrate profile in supporting various hormonal responses to modify nocturnal glucose metabolism may be shown by such clinical findings. Additional research has concentrated on the significance of carbohydrate consumption for the quality of sleep, with mounting data suggesting that the quality of carbohydrates in particular plays a significant role in determining the quality of sleep (30, 31). More specifically, reduced incidence of insomnia and improved quality of sleep have been associated with diets high in fiber and low in glycemic index carbohydrates (32). On the other hand, a higher frequency of sleep problems has been linked to diets heavy in starch, added sugars, and refined grains, or those with a high glycemic index (33, 34). However, whether LCD scores are associated with sleep disorders has not been reported. Our study suggests that RFM and a high-carbohydrate diet have an essential role in developing sleep disorders. Therefore, we believe that RFM and LCD scores are important in diagnosing sleep disorders and facilitating early disease recognition. This study is groundbreaking in examining the relationship between RFM and LCD score and sleep disorders.

The present study has several significant strengths. First, a cross-sectional design was used, employing a broadly representative sample from NHANES (National Health and Nutrition Examination Survey). The large sample size enhances the reliability and representativeness of the study. Second, the study utilized a complex multi-stage probability sampling design with extensive covariate adjustment. This approach improved the precision of statistical inference and provided plausible insights into the relationship between RFM and LCD scores and sleep disorders. Notably, our analyses also incorporated correlation analyses of RFM and LCD scores. Our results suggested that elevated LCD scores led to increased RFM and that those who were inherently obese also had a greater preference for a high-carbohydrate diet. Subsequently, we utilized a variety of regression models to explore the relationship between RFM and LCD scores and sleep disorders. In addition, we further showed that the effects of RFM and LCD scores on sleep disorders were robust by stratifying the analysis across different populations. Finally, we also conducted RCS analysis to explore whether there is a nonlinear relationship between RFM and LCD score and sleep disorders based on regression models incorporating multiple covariates. Our results revealed a significant effect model for the relationship between RFM and LCD score and sleep disorders, although this effect seemed to favor linearity over nonlinearity.

The present study also has some limitations. First, establishing a causal relationship between RFM and LCD scores and sleep disorders is challenging due to the cross-sectional design. Further longitudinal studies are needed to validate these findings. Second, data limitations in the NHANES database may have resulted in incomplete information on specific aspects of psoriasis, such as symptom severity and duration, which may affect the interpretation and accuracy of the results.

In summary, future longitudinal studies and more accurate data collection methods are warranted to more comprehensively explore the clinical manifestations of sleep disorders and their relationship with RFM and LCD scores. Such an exploration could provide more effective strategies for the intervention and prevention of sleep disorders.

In conclusion, our study found that RFM and LCD scores were associated with sleep disorders and explored subgroup differences with nonlinear relationships, thus providing important implications for clinical practice and public health. According to our study, diets that are high in fat and protein have been found to significantly contribute to the accumulation of relative fat mass, thereby playing a crucial role in the development of obesity. Furthermore, it has been observed that individuals with obesity who consume diets rich in fat and protein are at an elevated risk for experiencing sleep disorders. Since obesity with a LCD is a modifiable factor, it is possible to prevent and treat sleep disorders by promoting a healthy diet and appropriate exercise. However, further research is needed to determine whether RFM and LCD scores directly influence sleep disorders.

## Data Availability

The original contributions presented in the study are included in the article/supplementary material, further inquiries can be directed to the corresponding author.
